# Identification and Analysis of Long Repeats of Proteins at the Domain Level

**DOI:** 10.3389/fbioe.2019.00250

**Published:** 2019-10-08

**Authors:** David Mary Rajathei, Subbiah Parthasarathy, Samuel Selvaraj

**Affiliations:** Department of Bioinformatics, School of Life Sciences, Bharathidasan University, Tiruchirappalli, India

**Keywords:** long repeats, protein, domain, protein family, enzyme and non-enzyme classes, structural fold

## Abstract

Amino acid repeats play an important role in the structure and function of proteins. Analysis of long repeats in protein sequences enables one to understand their abundance, structure and function in the protein universe. In the present study, amino acid repeats of length >50 (long repeats) were identified in a non-redundant set of UniProt sequences using the RADAR program. The underlying structures and functions of these long repeats were carried out using the Gene3D for structural domains, Pfam for functional domains and enzyme and non-enzyme functional classification for catalytic and binding of the proteins. From a structural perspective, these long repeats seem to predominantly occur in certain architectures such as sandwich, bundle, barrel, and roll and within these architectures abundant in the superfolds. The lengths of the repeats within each fold are not uniform exhibiting different structures for different functions. We also observed that long repeats are in the domain regions of the family and are involved in the function of the proteins. After grouping based on enzyme and non-enzyme classes, we observed the abundant occurrence of long repeats in specific catalytic and binding of the proteins. In this study, we have analyzed the occurrence of long repeats in the protein sequence universe apart from well-characterized short tandem repeats in sequences and their structures and functions of the proteins at the domain level. The present study suggests that long repeats may play an important role in the structure and function of domains of the proteins.

## Introduction

Amino acid repeats are ubiquitous in protein sequences that often correspond to structural and functional units of proteins. The length of these repeats varies considerably from shorter units of homo repeats of single amino acid (Jorda and Kajava, 2010), oligopeptide repeats of 2–20 residues (Fraser and MacRae, 1973) and solenoid repeats of 20–40 residues to larger repetitions of length >50 called domain repeats (Andrade et al., 2001). These repeats occur as a single pair or as multiple copies in a tandem/non-tandem manner that are useful for structural packing or for one or more interactions with ligand (Katti et al., 2000; Luo and Nijveen, 2014). It has been observed that many proteins of length >500 contain internal repeats, suggesting the importance of repeats in producing larger proteins (Marcotte et al., 1998). However, these repeats possess weak identities due to extensive divergence, but retain similar folds and functions of the proteins (Holm and Sander, 1993). It has also been found out that long stretches of perfect repetitions are infrequent in protein sequences even though they are folded into recurrent structural motifs (Turjanski et al., 2016). Many methods and algorithms, such as Fourier transformation, short string extension, sequence-sequence alignment, and sequence profiles comparison have been introduced for the identification of such diverged sequence repeats with insertion and deletion without prior knowledge. Web based servers such as the Internal Repeat Finder, RADAR, REPRO, TRUST, XSTREAM, HHRepID, T-REKS, and PTRStalker (Pellegrini et al., 1999, 2012; George and Heringa, 2000; Heger and Holm, 2000; Szklarczyk and Heringa, 2004; Newman and Cooper, 2007; Biegert and Söding, 2008; Jorda and Kajava, 2009) have been developed by implementing the above techniques to detect amino acid repeats in proteins.

Earlier, proteins containing homo repeats (Jorda and Kajava, 2010), fibrous repeats (Fraser and MacRae, 1973) and different well-characterized repeats types, namely tetratricopeptide, leucine-rich, ankyrin and armadillo/heat etc. (Fraser and MacRae, 1973; Yoder et al., 1993; Groves and Barford, 1999; Kobe and Kajava, 2001), possessing different structures and functions have been analyzed (Andrade et al., 2001). Further, short units of repeats in tandem that form repeats in the structural folds of solenoids (α, β, α/β), β-trefoil (Murzin et al., 1992; Ponting and Russell, 2000), β-prisms (Chothia and Murzin, 1993; Bourne et al., 1999), and β-propellers (Bork and Doolittle, 1994; Neer et al., 1994) have been reviewed (Kajava, 2012). Recently, a detailed analysis and classification of β-hairpin repeat structures has been carried out (Roche et al., 2017). Also, it has been pointed out that short tandem repeats accumulate in the intrinsically disordered regions (IDR) (van der Lee et al., 2014) and play an important role in protein interactions and stability (Tompa, 2012; Habchi et al., 2014).

Analysis of larger proteins has demonstrated that significant portions of proteins are composed of domains. They are the conserved parts of proteins which can fold and function independently. The folded domains can either serve as modules for building up large assemblies or provide specific catalytic enzyme functions or bindings of the proteins. It has been found that repeats of a length >50 residues often correspond to conserved regions that are present in proteins as single or multiple copies for the function of the proteins (Hemalatha et al., 2007). Our analysis of sequence repeats of the proteins with known 3D structures in the PDB (Berman et al., 2014) has shown that they retain similar folds in spite of divergences, in order to conserve the structure and function of the proteins and, repeats that are in the single/two domains from the same family contain conserved motifs for the function of the proteins (Mary Rajathei and Selvaraj, 2013). Further, the conservation of inter-residues interactions in domain repeats have been analyzed in terms of long-range contact, surrounding hydrophobicity and pair-wise interaction energy (Mary et al., 2015). A database IR-PDB for repeats in the sequence of the proteins in the PDB has been developed for the analysis of impact of repeats in proteins (Selvaraj and Rajathei, 2017).

The widely used sequence database UniProtKB (UniProt Consortium T, 2017) contains more than 500,000 sequences that are annotated with well-characterized repeats of tetratricopeptide, leucine-rich repeats, ankyrin, and armadillo/heat etc. However, there has been no survey of repeats of length>50 in the UniProt sequences, which may provide insights into their role in the structure, function and evolution of the proteins. In the present study, we have analyzed the occurrence of long repeats and their underlying structures and functions in a non-redundant set of UniProt sequences. Since repeats of size exceeding 50 residues are large enough to fold independently into stable domains (Kajava, 2012), we used Gene3D for structural domains, Pfam for functional domains and enzyme and non-enzyme functions for specific catalytic and binding for their structure and function of long repeats proteins. It was found that long repeats occur in about 23% of the considered proteins. Analysis of the structure of long repeats reveals that these repeats are predominantly observed in the structural folds of sandwich, bundle, barrel and roll. We observed that repeats in the domains for the function of the proteins. Further, we observed that long repeats tend to occur both in enzyme and non-enzyme functions of proteins. While long repeats are found in all the major enzyme classes, these are more abundant among both ligases and isomerases. Among the non-enzyme proteins, such as DNA binding, metal binding, calcium binding, and Nucleotide binding (NP), these repeats are observed more in Nucleotide binding and DNA binding proteins. The present analysis shows that the occurrence of long repeats and their structures and functions of the proteins at the domain level.

## Materials and Methods

### Data Collection

**Figure 1:**
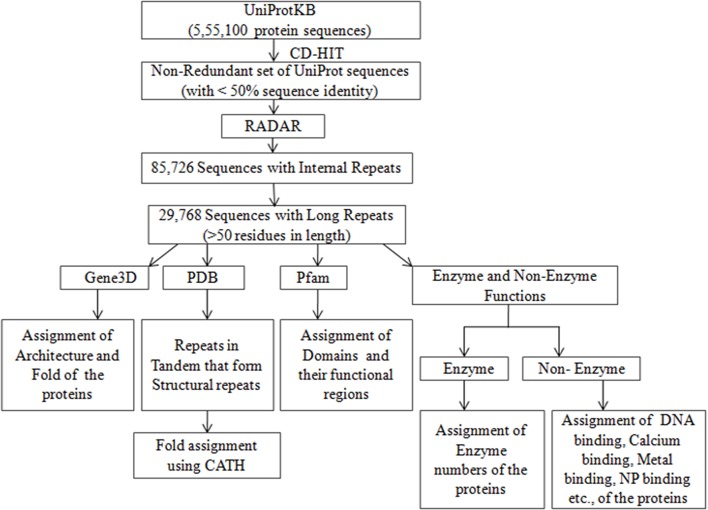
Flow diagram of identification and analysis of Long repeats from non-redundant set of UniProt sequences.

A collection of 555,100 proteins along with their assigned UniProt ID, amino acid sequence, protein name, protein family, enzyme function, and non-enzyme functions such as DNA binding, calcium binding, metal binding, and NP binding, as well as other annotation of the sequences from the databases of Pfam, Gene3D, PDB, and DisProt, was downloaded from UniProtKB/Swiss-Prot (UniProt Consortium T, 2017) and stored in a file. The Pfam is a database of protein domain families that assigns the domains, as well as their functional regions (Finn et al., 2014). Gene3D (Lewis et al., 2018), is a database that assigns the structure of the protein according to CATH hierarchy of class, architecture and fold in numerical values (Dawson et al., 2017). At the class level (C), the numerical value 1 is for all alpha class, 2 for all beta and 3 for a mixture of alpha and beta. Likewise, the numerical values are assigned for Architecture level (A) based on secondary structure arrangement in 3-D space and for Topology/Fold level (T) based on the connection of secondary structural elements. The PDB ID's of the 3D structure known proteins were obtained from the PDB database (http://www.rcsb.org/pdb/home/home.do). The intrinsic disordered regions of the proteins that were extracted from the literature are available in the DisProt database (Piovesan et al., 2017). A non-redundant representative set of 126,945 sequences that share <50% sequence identity was obtained by clustering the 555,100 sequences using the web server CD-HIT (Fu et al., 2012). The overall work-flow is summarized as a flowchart (Figure 1).

### Finding Sequence Repeats of the Proteins Using RADAR

The presence of internal repeats in each protein sequences was identified using the repeat detection program RADAR (Heger and Holm, 2000), which was downloaded from the URL (https://sourceforge.net/projects/repeatradar). The RADAR program is efficient for *ab initio* detection of repeats of length >15 in a single sequence by aligning the sequence against itself, as well as by generating the sequence profile using multiple sequence alignment. RADAR evaluates the statistical significance of the observed repeats by measuring a Z-score for each repeat unit (McLachlan, 1983; Heringa and Argos, 1993). The *Z*-score of a repeat unit is the number of standard deviations of the repeat unit score above the mean. The score of each unit is determined from a profile derived from the multiple alignment of repeat unit without considering end-gaps. Repeats with *Z*-scores threshold of > 6 are reported by the RADAR program. An in-house Perl program that incorporated the RADAR executable was written to detect internal repeats of all sequences in the dataset in a single run. Proteins containing repeats of length >50 were considered for further analysis.

### Finding the Structure of Long Repeats Proteins

The UniProt ID's of proteins having long repeats were extracted and their Gene3D structural domain-based assignments of the proteins were extracted using a Perl program. Then, the name of class, architecture and fold of the protein was found out by using CATH search and grouped according to their name for the further analysis of architecture and fold of the protein with repeats.

### Finding the Functional Domains of Long Repeats Proteins

The UniProt ID's of long repeat proteins were extracted and their assigned Pfam domains of the sequences were identified. The domain regions and their functional residues information of the proteins were found out using Pfam database search (Finn et al., 2014), and repeats in the domain regions were identified by manual search. The level of similarity of the repeats within a protein and within a protein family was found out in terms of % sequence identity through using the Needleman–Wunsch algorithm (Needleman and Wunsch, 1970) implemented in the ggsearch36 program of the FASTA-36.3.5b package (Henikoff and Henikoff, 1992). Needleman–Wunsch alignment scores were calculated using the BLOSUM50 scoring matrix (Pearson, 2000) with a penalty of −12 for gap opening and −2 for gap extension. Further, the repeats in domains of the proteins were also analyzed for their functional involvement at the structure of the proteins using the server PDBsum by giving PDB ID as input (Laskowski et al., 2018).

### Finding the Enzyme and Non-enzyme Functions of Long Repeats Proteins

The assigned enzyme numbers (EC) of long repeats proteins were extracted. The EC number of the protein at the first level corresponds to seven enzyme classes of Oxidoreductases (EC 1), Transferases (EC 2), Hydrolases (EC 3), Lyases (EC 4), Isomerases (EC 5), Ligases (EC 6), and finally, Translocases (EC 7). The enzyme numbers were extracted and grouped according their numbers for further analysis. The non-enzyme proteins that are assigned with DNA binding, calcium binding, metal binding and NP binding were also extracted and grouped according to their name.

## Results

### Abundance of Proteins Having Long Repeats

**Figure 2:**
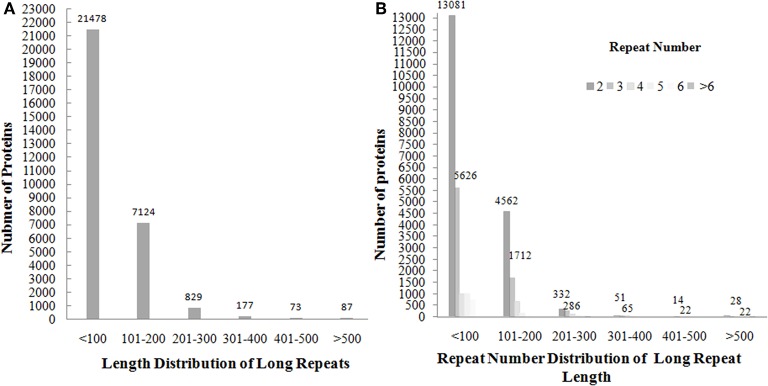
The plotting of number of proteins against the distribution of long repeats of length >50 in the range of <100, 101–200, 201–300, 301–400, 401–500, and>500 shows that most of the longrepeat lengths fall in the range of <200 **(A)** and repeat number distribution of long repeats shows that repeat numbers of 2 and 3 in most of long repeat proteins **(B)**.

The presence of amino acid repeats of length >15 was found out in 85,726 (67%) out of non-redundant set of 126,945 UniProt protein sequences (Supplementary Data File 1). The long repeats were found out in 29,768 (35%) proteins. These repeats are present as a single pair or multiple copies of repeats in tandem/non-tandem manner. For example, N-acetylmuramoyl-L-alanine amidase Rv3717 protein (UniProt ID: I6Y4D2) of length 241 contains a single pair of tandem repeats of length 96 in the continuous region of 12–116/118–226. Complement control protein C3 (P68639) of length 263 has three copies of repeats of length 55 in tandem (81–142/143–200/201–254), whereas Transcriptional regulatory protein TyrR (UniProt ID: P44694) of length 318 contains a single pair of non-tandem repeats of length 76 in the discontinuous region of (19–99/200–279). The length of the repeats varies in the range of 51–1759 and lengths of >1,000 are mostly found out in enzyme proteins. For example, the non-ribosomal peptide synthetase 1 (Q4WT66) of sequence length 6,269 contains repeats of length 1,546 (277–905/906–2,334/2,335–3,465/3,466–4,590/4,593–5,634). Through the analysis of length distribution of long repeats, as well as their repeat number distribution of long repeats against the number of proteins (Figure 2), we observed that the lengths <200 are observed in more than 90% of the proteins with an average of 100 residues, and repeated in 2–5 number of times with repeat numbers of 2 (61%) and 3 (26%) in most of the long repeat proteins. The *Z*-score values of the repeats were extracted and found that 74,089 out of 74,154 repeat units have *Z*-scores >6. Among these, 66,400 repeat units have *Z*-scores of >20. This suggests that most of the observed repeats are statistically significant.

### Analysis the Structure of Long Repeats Proteins

The structural class, architecture and fold of the 14,176 proteins (48%) have been found out using structural domain based Gene3D assignments. Among these, some proteins are having two or more Gene3D assignments. In this study, 10,504 proteins that contained a single Gene3D assignment were considered for further analysis (Supplementary Data File 2). For example, Annexin A1 (P04083) protein contains a single Gene3D assignment of 1.10.220, which means that this protein belongs to class alpha (1) of orthogonal bundle architecture (10) with Annexin V domain fold (220).

### Analysis of Long Repeats at the Architecture Level

**Figure 3:**
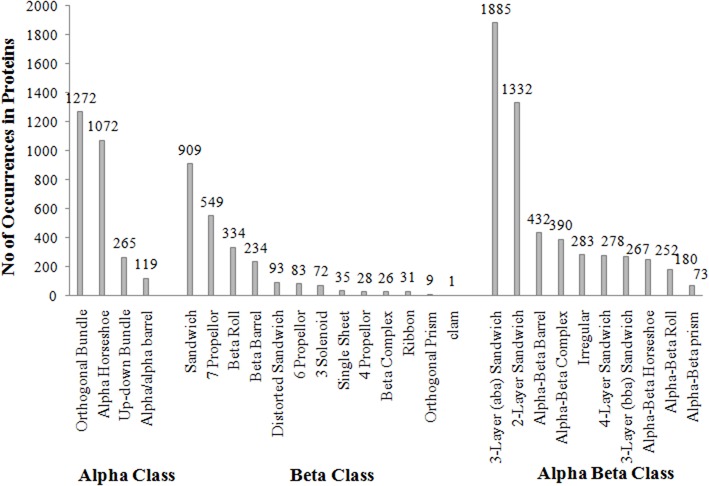
Number of Long repeats containing proteins assigned with different architectures of α, β, and α/β class using CATH.

According to CATH domain-based hierarchy (http://www.cathdb.info/browse/tree), the presence of long repeats in different architectures of alpha (α), beta (β), and alpha/beta (α/β) class proteins was observed (Figure 3). Out of five architectures of α class, these were observed in the four architectures, namely orthogonal bundle, up-down bundle, α horseshoe and α/α barrel. Among these, substantial numbers were present in the architectures of bundle and horseshoe. Under β class, the repeats were present in 13 out of 20 architectures and the sandwich, propeller, roll and barrel were observed most. Likewise, repeats were found in 10 out of 14 architectures of α/β class and the architectures of 3-layer (αβα) sandwich, 2-layer sandwich, α/β barrel and αβ-complex were observed most. By combining the architectures from different classes of proteins, repeats in specific architectures of sandwich, bundle, barrel and roll compared to other architectures were found out.

### Analysis of Long Repeats at the Fold Level

**Figure 4:**
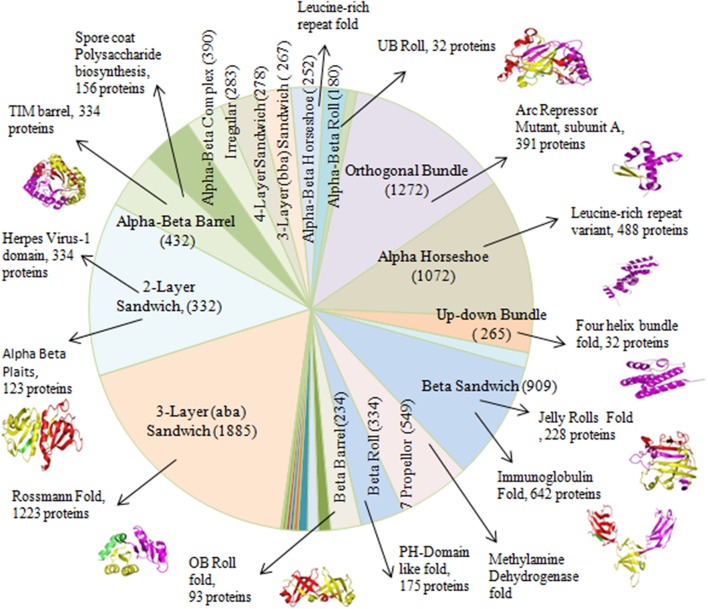
The occurrences of certain folds of Arc Repressor Mutant subunit A of Orthogonal bundle architecture, Four helix bundle of Up-down bundle, Jelly Rolls and Immunoglobulin of Beta sandwich, PH-Domain like fold of Beta Roll, OB Roll of Beta Barrel, Rossmann fold of 3-layer sandwich, Alpha Beta plaits, and Herpes Virus-1 domain of 2-layer sandwich, TIM barrel of Alpha-Beta Barrel and UB Roll of Alpha-Beta Roll in a substantial numbers of Long repeats proteins.

The existence of repeats in different folds of sandwich, bundle, barrel and roll architectures was found out. Repeats were observed in 84 out of 287 folds in orthogonal bundle and 32 out of 101 folds in up-down bundle of α class. At the β class, 12 out of 43 folds in β sandwich, 18 out of 48 folds in β barrel, and 13 out of 40 folds in β roll architecture of the proteins were having repeats. Under α/β class, repeats in 47 out of 126 folds under 3-layer (αβα) sandwich, 57 out of 224 under 2-layer sandwich, 6 out of the 18 folds under α/β barrel and 16 out of 58 folds under α/β roll were observed. Among that, some folds were observed in a greater number of proteins compared to other folds (Figure 4). In α class, the Arc Repressor Mutant Subunit A fold and four Helix Bundle fold of bundle architectures were observed in most of the proteins compared to other folds (Table 1). Under β class, the Immunoglobulin-like fold and Jelly Roll fold of β-sandwich, PH-domain fold of β-roll, and OB fold of β-barrel were observed most. The Rossmann fold in 3-layer (αβα) sandwich, TIM Barrel in αβ barrel and Herpes Virus-1 followed by Alpha-Beta plaits fold of 2-layer Sandwich, and Ubiquitin-like (UB roll) of αβ roll were observed most. The results reveal the predominant occurrence of long repeats in the diverse structure exhibiting folds of the proteins.

**Table 1:** Number of proteins containing long repeats in the architectures and folds of the proteins.

**Class**	**Architecture**	**Number of proteins**	**Fold**	**Number of proteins**
Alpha (α) class	Orthogonal bundle	1,272	Arc repressor mutant, subunit A	391
	Alpha horseshoe	1,072	Leucine-rich repeats variant	488
	Up-down bundle	265	Four helix bundle	32
Beta (β) class	Beta sandwich	909	i)Jelly Rolls ii)Immunoglobulin	228 642
	7 Propeller	549	Methylamine dehydrogenase	549
	Beta roll	334	PH-domain like	175
	Beta barrel	234	OB Roll	93
Alpha Beta (αβ) class	3-Layer (aba) Sandwich	1,885	3-layer(αβα) sandwich	1,223
	2-Layer sandwich	1,332	Alpha beta plaits	123
	Alpha-beta barrel	432	TIM barrel	334
	Alpha-beta complex	390	Spore coat polysaccharide biosynthesis protein SpsA	156
	Alpha-beta roll	180	UB Roll	32

### Analysis of Long Repeats for Structural Repeats

**Figure 5:**
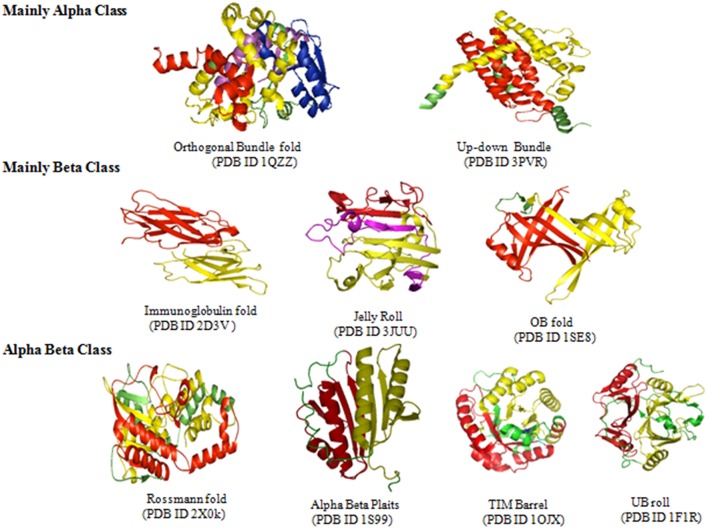
Long repeats that form structural repeats in the folds of Orthogonal bundle, Up-down bundle of alpha class, Immunoglobulin fold, Jelly Roll, OB fold of beta class, Rossmann fold, Alpha Beta Plait, TIM barrel, and UB roll of alpha-beta class.

**Figure 6:**
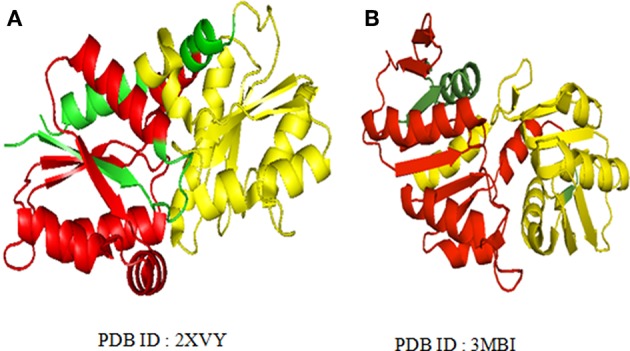
Varying larger repeat lengths are observed in the Rossmann fold of the **(A)** Cobalt chelatase CbiK (2xvyA) with repeat length 103 and **(B)** Phosphoribosyl pyrophosphate synthetase (3 mbiA) with repeat length 121. The repeats regions are highlighted with different colors.

The long repeats in proteins with known 3-D structure (as available from UniProt annotation) were analyzed for structural repeats. The proteins with tandem repeats were found out and analyzed at the structural level. We observed long tandem repeats form structural repeats in the folds of up-down and orthogonal bundle of α-class, Immunoglobulin, Jelly Roll and OB fold of β-class, Rossmann fold, TIM barrel, α/β plait, and UB roll of α/β class. Figure 5 shows the structural repeats of the proteins in the folds of up-down and orthogonal bundle of α-class, Immunoglobulin, Jelly Roll and OB fold of β-class, Rossmann fold, TIM barrel, α/β plait, and UB roll of α/β class. Further, we found out that the lengths of the repeats are not uniform and vary considerably within each fold. Figure 6 shows the considerable variation in lengths, as well as in the secondary structures of different proteins possessing the Rossmann fold that usually contains βαβαβ secondary structure arrangements. The Desulfovibrio vulgaris CbiK(P) Cobaltochelatase (PDB ID: 2XVY) contains two repeats of βαβαβαβ secondary structure arrangement of length 103 (Figure 6A) (Malay et al., 2009), whereas, another protein Thermoplasma volcanium Phosphoribosyl pyrophosphate synthetase (PDBID: 3MBI) contains two repeats of βαβαβαβα of length 121 in Figure 6B (Cherney et al., 2011). The analysis results suggest that the length variations of repeats within the Rossmann fold lead to the presence of additional α-helices, β-strands, and coil regions. Thus, longer repeats of different lengths provide the structural differences within a fold of the proteins.

### Analysis of Long Repeats for Intrinsic Disordered Region

The intrinsically disordered regions (IDR) for 51 (<1%) of long repeats proteins were found out using DisProt database. While analyzing the predisposition of long repeats for IDR, most of the repeats were identified in the structured regions. However, we also identified long repeats in an IDR. For example, Nucleoporin NUP1 (P20676) protein of length 1,076 contains tandem repeats of length 62 in the region of (352–399/403–462/522–564/666–728/731–778/779–840/849–906/907–972/978–1,031), which has been identified as an IDR (300–1,078). This analysis suggests that long repeats are generally structured in most of the proteins while few of them may have IDRs.

### Analysis of Functions of Long Repeats at the Domain Level

**Figure 7:**
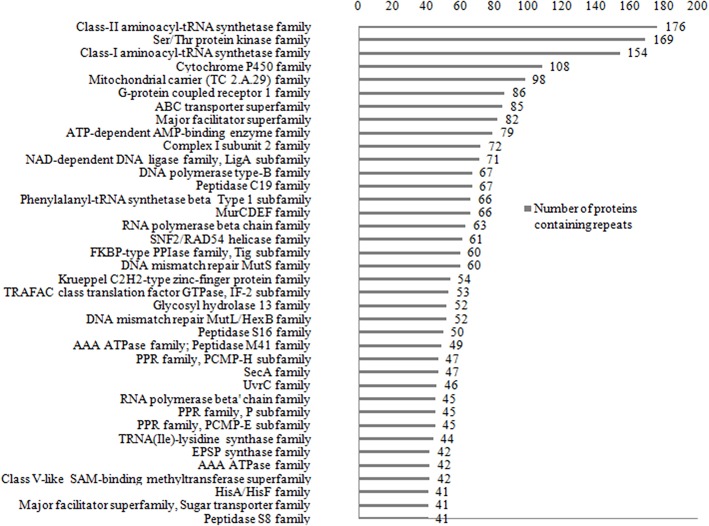
List of the 36 protein families that are having long repeats in more than 40 member proteins.

**Figure 8:**
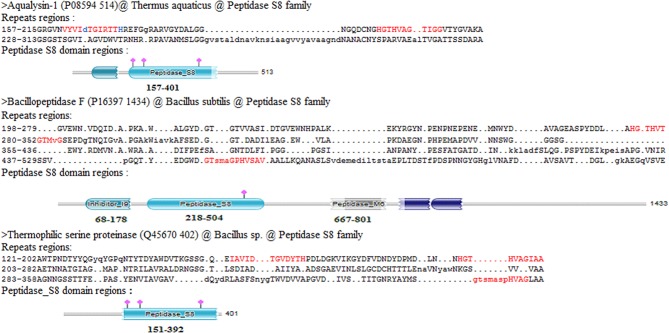
The Aqualysin-1(P085594), Bacillopeptidase F(P16397), and Thermophilic serine proteinase (Q45670) protein's repeats regions and their alignments that are in the Peptidase S8 domain region assigned by Pfam.

The Pfam domain assignments in 26,750 (90%) of proteins were found and suggested the occurrence of repeats in the functional domain families containing proteins. While grouping by protein family, the existence of repeats in 5,258 distinct protein families was found out. Some of the protein families are having long repeats in a greater number of their member proteins (Supplementary Data File 3). Figure 7 shows the list of 36 protein families such as Class II aminoacyl-tRNA synthetase, Ser/Thr Protein kinase, Class I aminoacyl-tRNA synthetase, Cytochrome P450, Mitochondrial carrier (TC 2.A.29), G-protein coupled receptor 1, and ABC transporter that are having repeats in more than 40 member proteins of the family. We observed long repeats in the domains of the family with varying lengths. For example, the Peptidase S8 family proteins contained long repeats in 41 member proteins of the family (Table 2). Among these, 38 protein repeats were in the Peptidase S8 domains with varying repeat lengths. Figure 8 shows some of the proteins' repeat regions as well as their alignment that covers the Peptidase S8 domain regions. The level of similarity between the repeats in the Peptidase S8 domain within a protein and within the member proteins of the Peptidase S8 domain family was computed in terms of % sequence identity. For example, the sequence identity of 29% was observed for the repeats (157–215/228–313), within the Peptidase domain (157–401) of the Aqualysin-1 protein (P08594) (Table 2). Further, the sequence similarities of repeat unit (157–215) of this protein, with the repeat units in the Peptidase S8 domain of the 37 member proteins, were also computed. We observed that 65 % of repeats were in the range of 20–40% sequence identity and the remaining protein repeats were in the range of 10–20% identity. This observation suggests that the repeats within a protein, as well as within a protein family, are considerably diverged.

**Table 2:** List of 41 member proteins of the Peptidase S8 family long repeat region's and their function domain regions assigned using Pfam.

**S. NO**.	**Protein name (UniProt ID and length)**	**Long repeats regions and their length**	**Peptidase S8 domain regions and their length**	**Other domains and their regions**
1	Aqualysin-1 (P08594 514)	157–215/228–313 (57)	157–401 (244)	Inhibitor_I9 (54–125)
2	Bacillopeptidase F (P16397 1434)	i)198–279/280–352/355–436/437–529 (85) ii)568–609/615–701/1,044–1,167 (79)	218–504 (286)	Peptidase_M6 (667–801) Inhibitor_I9 (68–178)
3	Calcium-dependent protease (Q59149 663)	219–313/315–412 (93)	228–530 (302)	P_proprotein (547–662)
4	Cell wall-associated protease (P54423 895)	737–803/818–885 (67)	458–729 (271)	
5	Cuticle-degrading protease (P29138 389)	72–171/172–270/277–355 (84)	139–383 (244)	Inhibitor_I9 (41–107)
6	Extracellular serine protease (P29805 1046)	i)158–240/241–381/385–491 (137) ii)509–585/586–685/687–754/771–831 (83)	71–397 (326)	Autotransporter (769–1,045)
7	Microbial serine proteinase (P31339 622)	167–237/389–458 (67)	89–411 (322)	P_proprotein (491–572)
8	Minor extracellular protease vpr (P29141 807)	18–148/149–208/340–473/475–532/658–711 (183)	184–594 (410)	Inhibitor_I9 (57–143); PA superfamily (355–497); FlgD_ig superfamily (712–792)
9	Minor extracellular protease Epr (P16396 646)	39–112/169–240/249–327 (74)	137–380 (243)	
10	MycP4 protease (I6YC58 456)	25–208/222–407 (159)	86–389 (303)	
11	MycP1 protease (A0QNL1 450)	91–172/173–327/332–428 (127)	83–381 (298)	
12	Nisin leader peptide-processing serine protease (Q07596 683)	228–281/379–418/504–557 (52)	255–546 (291)	
13	PIII-type proteinase (P15292 1963)	156–206/208–209/295–380 (82)	212–698 (486)	
14	Proprotein convertase subtilisin/kexin type 9 (Q80W65 695)	467–537/540–611/616–682 (140)	185–423 (238)	Inhibitor_I9 (80–152)
15	Pyrolysin (P72186 1399)	i)225–274/276–339/341–400 (62) ii)959–1,006/1,011–1,158/1,169–1,296 (126)	i)174–380 (206) ii)408–654 (246)	
16	Putative subtilisin-like proteinase 1 (Q8SQJ3 466)	23–92/94–160/165–195 (67)	144–422 (278)	Inhibitor_I (919–90)
17	Putative subtilisin-like proteinase 2(Q8SS86 536)	106–165/278–336/362–390 (60)	272–452 (180)	
18	Probable subtilase-type serine protease DR_A0283 (Q9RYM8 729)	84–126/131–209/232–310/320–378 (78)	183–470 (287)	Peptidase_M14NE-CP-C_like(486–558); PPC (624–693)
19	Subtilase-type proteinase psp3 (Q9UTS0 452)	217–283/349–407 (56)	202–429 (227)	Inhibitor_I9 (80–162)
20	Subtilase-type proteinase RRT12 (P25381 492)	53–107/269–320 (52)	156–389 (233)	
21	Subtilisin-like protease SBT3.13 (Q8GUK4 767)	320–431/608–719 (107)	153–588 (453)	Inhibitor_I9 (41–119); PA_Superfamily (384–485)
22	Subtilisin-like protease SBT4.4 (Q9FGU3 742)	65–226/416–581 (150)	137–581 (444)	Inhibitor_I9(34–112); PA(338–458)
23	Subtilisin-like protease SBT4.10 (Q9FIM8 694)	138–284/387–534 (139)	138–526 (388)	Inhibitor_I9 (35–113);PA (332–371)
24	Subtilisin-like protease SBT4.14 (Q9LLL8 750)	202–333/336–464/467–596 (129)	141–594 (453)	Inhibitor_I9 (38–115); PA (346–467)
25	Subtilisin-like protease SBT2.4 (F4HYR6 833)	245–361/362–547/548–736 (178)	169–691 (522)	Inhibitor_I9 (70–138);PA (389–533)
26	Subtilisin-like protease SBT4.15 (Q9LZS6 767)	284–379/450–550 (92)	137–590 (453)	Inhibitor_I9 (35–113); PA (342–474)
27	Subtilisin-like protease SBT3.18 (Q9STQ2 780)	i)179–224/495–575/707–756 (76) ii)318–388/405–476 (65)	137–613 (476)	Inhibitor_I9 (30–109); PA_Superfamily (361–482)
28	Subtilisin-like protease SBT6.1 (Q0WUG6 1039)	556–644/812–901 (86)	208–486 (278)	
29	Subtilisin-like protease SBT2.2 (Q9SUN6 857)	163–222/226–283 (54)	184–674 (490)	Inhibitor_I9 (98–159); PA superfamily (406–548)
30	Subtilisin-like protease SBT2.6 (Q9SZV5 817)	155–186/195–224/315–398 (59)	151–635 (484)	Inhibitor_I9 (61–124); Pasuperfamily (374–511); fn3_5 superfamily (698–810)
31	Subtilisin-like protease SBT3.6 (Q8L7I2 779)	216–306/307–392 (71)	138–593 (455)	Inhibitor_I9 (34–113); PA superfamily (365–493)
32	Subtilisin-like protease SBT1.2 (O64495 776)	150–259/527–633 (101)	127–587 (460)	Inhibitor_I9 (27–112); PA superfamily (353–481)
33	Subtilisin-like protease SBT1.4 (Q9LVJ1 778)	169–268/435–532 (86)	133–589 (456)	Inhibitor_I9 (32–110); PA superfamily (355–474)
34	Serotype-specific antigen 1 (P31631 933)	378–433/466–588/594–709 (115)	54–408 (354)	Autotransporter superfamily (673–916)
35	Subtilisin-like protease 12 (D4AQA9 417)	253–300/305–372 (64)	145–399 (254)	Inhibitor_I9 (35–116)
36	Subtilisin-like protease CPC735_047380 (C5PFR5 401)	84–138/140–196 (53)	143–363 (220)	Inhibitor_I9 (35–114)
37	Tripeptidyl-peptidase 2 (Q09541 1375)	370–469/688–782 (87)	89–559 (470)	TPPII (832–1,017)
38	Tripeptidyl-peptidase 2 homolog (Q9UT05 1275)	i)265–379/413–522 (96) ii)637–807/820–958 (121)	90–545 (450)	TPPII (837–1,008)
39	Thermophilic serine proteinase (Q45670 402)	121–202/203–282/283–358 (79)	151–392 (241)	
40	Tripeptidyl-peptidase 2 (F4JVN6 1381)	i)143–207/343–403/717–760 (62) ii)1,043–1,188/1,236–1,380 (135)	140–620 (480)	TPPII (897–1,078) SMC_N (1,140–1,355)
41	Subtilisin-like protease (Q00139 371)	20–72/80–135 (52)	83-255 (172)	P–proprotein (240–370)

**Figure 9:**
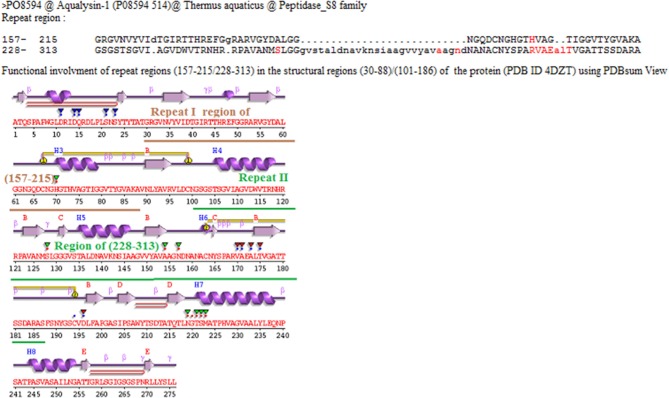
The functional residues of repeats (157–215/228–313) in the structural regions (30–88)/(101–186) (highlighted by red and green inverted triangles with red color dots) of Aqualysin-1 proteins are found out using PDBsum search.

Further, repeats in the domains are involved in the function through functional residues (highlighted in red color). For example, the regions (162–173) and (197–207) of repeats (157–215/228–313) of Aqualysin-1 (UniProt ID P08594) have contained functional residues VYVIDTGIRTTH and HGTHVAGTIGG for Serine proteases (Figure 8). The functional involvement of the repeats was also found out in the structure of the proteins using PDBsum. For example, the functionally involved residues (highlighted red in color) of repeats (157–215/228–313) in the structure of Aqualysin-1 (PDB ID 4DZT) were found out using PDBsum search (Figure 9). This suggests that these repeats occur in the domains of the family for the function of the proteins.

### Analysis of Enzyme and Non-enzyme Functions of Long Repeats

Further, the enzyme functions in 13,333 proteins and non-enzyme functions in 2,437 proteins, of a total of 15,770 (53%) of long repeats proteins, were also found out. Of a total of 13,333 enzymes having long repeats, Ligases (35.91%) have the maximum number of repeats followed by Isomerases (28.98%), Translocases (11.81%), Transferases (11.12%), Lyases (5.12%), Hydrolases (4.74%), and Oxidoreductases (2.32%). Among the non-enzymes in 2,437 proteins, NP binding proteins (48.09%) have the maximum number of repeats followed by DNA binding (30.44%), metal binding (16.94%), and calcium binding (4.51%). These observations suggest the importance of long repeats in both the catalytic and binding function of proteins apart from serving as modules of large assemblies.

## Discussion

Our survey of long repeats in a non-redundant set of UniProt sequences has highlighted the occurrence of these repeats that play an important role in the structure and function of domains of the proteins. Previous studies have focused on structural and functional implications of proteins with homo repeats (Uthayakumar et al., 2012), fibrous repeats (Parry, 2005) and different well-characterized repeats of length 5–50 (Andrade et al., 2001). Therefore, an in-depth study of long repeats in UniProt sequences was carried out for a better understanding of the correspondence of repeat sequences with their structures and functions. In this study, we used the RADAR program for internal repeat detection, since it often detects both tandem and interspersed repeats in larger size. Our earlier studies for repeats analysis (Mary Rajathei and Selvaraj, 2013; Mary et al., 2015) have shown the ability of RADAR to detect repeats of length > 50 that are structurally similar and conserved in a 3D structure environment. Further, the sensitivity and accuracy of RADAR repeats, by comparison with Pfam, indicate good coverage, accurate alignments, and reasonable repeat borders (Heger and Holm, 2000). The identified repeats vary in the range of 50–1,759 of lengths and diverged with more insertions and deletions, but the calculated z-scores by RADAR have shown their statistical significance.

From a structural perspective, long repeats tend to occur abundantly in certain architectures of sandwich, barrel, bundle, and roll. Within these architectures, they are predominately observed in the super folds of up-down and orthogonal bundle of α-class, Immunoglobulin, Jelly Roll and OB fold of β-class, Rossmann fold, TIM barrel, α/β plait, and UB roll of α/β class of the proteins. The adoption of classic super secondary elements (αα, βαβ, ββ) and incorporation of repetitive duplication of a small stable unit may be the possible reasons for abundance of larger duplication in these folds (Thornton et al., 1999). For example, the evolution of the (βα)_8_ repeat in the TIM barrel is through repetitive duplication of a small stable unit (βα) (Lang et al., 2000). It has been observed that repeats in the folds may fulfill the physical demand (stable and fast folding conformation) of the protein chain during the process of evolution, in order to meet the cellular function (Lupas et al., 2001). Further, it has been shown that the existence of structural symmetries in the super-folds (6 out of 10) may also require larger duplication during evolution of the proteins (Brych et al., 2003). Kim et al. (2010), through their SymD (detecting symmetry in protein structures) method, have identified 33 folds that contain 10 or more symmetric domains. There is considerable overlap between the symmetry in the folds they identified and those observed in the present work (Figure 5). We observed that long repeats of different lengths within a fold provide the structural differences of the proteins for different functions. Further, the analysis of predisposition of long repeats for disordered regions has shown that long repeat proteins are mostly structured to form stable folds. However, it has been observed that short tandem repeats are highly disordered, which do not adopt a single defined configuration for specific function (Tompa, 2012; Habchi et al., 2014; van der Lee et al., 2014).

Further, repeats have been analyzed for a specific domain of the family, in which protein function could be found out through the domain (Rentzsch and Orengo, 2013). We found that repeats in the domain regions of the family are involved in the function through functional residues. Earlier, we analyzed the repeats in the individual proteins of PDB and found that the existence of repeats in single/two domains from the same family, for the function of the proteins and that are not in the domains, are also involved in the function of the proteins (Mary Rajathei and Selvaraj, 2013). We observed that the lengths of repeats in the domains of the family are not uniform. Further, the computation of sequence identity of the repeats within a protein and within a family of Peptidase S8 domain shows lower similarity, which may be the consequence of their divergences over a period. Earlier, it was observed that repeat proteins are indeed repetitive in their families, exhibiting abundant stretches of short perfect repetitions (Turjanski et al., 2016). The repeats of varying lengths in the structures of the fold, as well as in the functional domains of the family, have suggested that long repeats are considerably diverged and may not be overlapped. However, further studies would be needed to understand the conservation of long repeats of the proteins in the structure and function of the proteins.

Further, we observed the existence of long repeats in all seven enzyme classes of the proteins and are especially more abundant in ligases and isomerases. Among the non-enzyme proteins, long repeats are observed in DNA binding, calcium binding, metal binding and NP binding proteins with NP binding and DNA binding in a greater number of proteins. However, further studies are needed to understand why certain enzyme classes and non-enzyme classes are having long repeats in more numbers. This shows that the occurrence of long repeats, not only serves as modules of large assemblies, but also in the catalytic function or binding of the proteins.

While commenting on the evolution of the well-characterized short tandem repeats in many evolutionary lineages, it has been postulated that repeat-containing proteins are cheap to evolve, rather than the *de nova* sequence evolution, as the repeat units are thermodynamically stable (Andrade et al., 2001; Andersson et al., 2015). Through our analysis, we observed the occurrence of long repeats in the stable folds for different functions of the proteins and suggested that long repeats may play a role in the evolution of proteins with stable folds and novel functions.

## Conclusions

The present large scale study has focused on the presence of long repeats in a non-redundant set of the entire annotated UniProtKB/Swiss-Prot database and reveals that long repeats are found in 23% of the proteins. Regarding their three-dimensional structures, they are found in certain structural folds that are incorporated with repetitive duplication of small stable folds. Further, the long repeats of different lengths within each fold are observed in different structures of the proteins. From a functional perspective, these repeats are found in both enzyme and non-enzyme functions containing proteins. Hence, long repeats may have a role in the evolution of proteins with stable folds and novel functions.

## Data Availability Statement

The UniProt annotated sequence files and the RADAR output files were analyzed for this study. Major results are available as Supplementary Material.

## Author Contributions

DR developed the computer programs in perl platform for this study and drafted the manuscript. SP supported to analyze and computation the data. SS conceived the idea and helped in the preparation of the manuscript.

### Conflict of Interest

The authors declare that the research was conducted in the absence of any commercial or financial relationships that could be construed as a potential conflict of interest.
